# Sex differences in the association between self-rated health and high-sensitivity C-reactive protein levels in Koreans: a cross-sectional study using data from the Korea National Health and Nutrition Examination Survey

**DOI:** 10.1186/s12955-020-01597-5

**Published:** 2020-10-14

**Authors:** Se-Won Park, Seong-Sik Park, Eun-Jung Kim, Won-Suk Sung, In-Hyuk Ha, Boyoung Jung

**Affiliations:** 1 Department of Sasang Constitutional Medicine, Dongguk University Bundang Oriental Hospital, Seongnam-si, Gyeonggi-do, Republic of Korea; 2 Department of Acupuncture and Moxibustion, Dongguk University Bundang Oriental Hospital, Seongnam-si, Gyeonggi-do, Republic of Korea; 3 Jaseng Spine and Joint Research Institute, Jaseng Medical Foundation, Seoul, Republic of Korea; 4 Department of Health Administration, Hanyang Women’s University, 200, Salgoji-gil, Seongdong-gu, Seoul, 04763 Republic of Korea

**Keywords:** High-sensitivity C-reactive protein, Korea national health and nutrition examination survey, Self-rated health, Cross-sectional study

## Abstract

**Background:**

No studies have investigated the association between self-rated health (SRH) and high-sensitivity C-reactive protein (hs-CRP) levels in South Koreans. We explored this association and analyzed differences between sexes.

**Methods:**

Using cross-sectional data from the 2015–2017 Korea National Health and Nutrition Examination Survey, we analyzed the association between SRH and high hs-CRP levels (> 1.0 mg/L) in 14,544 Koreans aged ≥ 19 years who responded to the SRH survey and had available hs-CRP test results. Differences in sociodemographic factors were analyzed using the Pearson’s chi-square test for categorical variables or the Mann–Whitney U test for continuous variables. Multiple logistic regression analysis was used to measure the association between hs-CRP levels and SRH according to sex while adjusting for other possible confounders.

**Results:**

The percentage of very poor to poor SRH was higher in the high hs-CRP group (22.4%) than in the low hs-CRP group (17.66%). Among men, the risk of a high hs-CRP level increased with worse SRH (adjusted for confounders; P for trend < 0.001). After adjusting for all confounders, including chronic diseases, men with very poor SRH showed a higher odds ratio (OR) for high hs-CRP levels than those with very good SRH (fully adjusted OR, 1.74; 95% CI, 1.04–2.90). Significant correlations were absent among women.

**Conclusions:**

Poor SRH was correlated with low-grade inflammation (high hs-CRP levels) among Korean male adults. These findings could be useful for developing health improvement programs and in goal setting at a national scale.

## Background

Self-rated health (SRH) is an index utilized worldwide to summarize how patients perceive their overall health status [1]. SRH is an independent predictor of mortality and disease morbidity, even after adjusting for demographic, sociological, and medical risk factors [2]. Despite criticisms that SRH is assessed based on a single question, it is known to be a strong predictor in both healthy and unhealthy individuals. SRH is not only a predictor of previously diagnosed disease but also a predictor of reactions associated with the progression of disease in the premorbid stage; it encapsulates recent or sporadic health issues that may be missed by one-time objective testing, and it also reflects behavioral and emotional factors [2, 3].

C-reactive protein (CRP) is produced by hepatocytes following acute tissue injury or infection. Though CRP levels are generally elevated in cases of severe inflammation, high-sensitivity CRP (hs-CRP) levels increase nonspecifically in the event of inflammation in the body. In particular, hs-CRP is used as an indicator to assess the risk of cardiovascular disease (CVD), and several studies have suggested hs-CRP as a predictor of mortality. In assessing CVD risk, the American Heart Association (AHA) and Centers for Disease Control and Prevention (CDC) defined hs-CRP levels of > 3.0 mg/L to indicate high risk, 1.0–3.0 mg/L to indicate average risk, and < 1.0 mg/L to indicate low risk [4, 5].

In South Korea, the percentage of individuals who consider their health status to be good (“very good” or “good”) is low, at 29.2% in 2017. From 1998 to 2017, this percentage has remained in the range 29–47%, indicating that less than half of the population consider themselves to be healthy. Moreover, this percentage is one of the lowest among countries of the Organization for Economic Co-operation and Development; even though South Korea maintains an objective health status similar to that of the US and Europe, South Koreans experience more subjective health anxiety [6].

Both SRH and CRP have been used as indicators of mortality and morbidity, and several studies have reported a relationship between them [7–9]. Shanahan et al. [8] suggested that an elevated hs-CRP level is associated with poor SRH. Conversely, other studies have suggested that poor SRH is associated with elevated hs-CRP levels [10]. Moreover, other studies have reported an association between albumin levels, hemoglobin levels, white blood cell counts, and HDL-cholesterol levels [11].

The level of inflammation differs according to sex and genetic variation [12, 13]. However, previous studies have reported inconsistent results for sex-based differences in the association between SRH and CRP levels. Some studies found a significant association between SRH and low-grade inflammation only in men [8, 14], whereas one study demonstrated opposite results [9]; others suggested that the association is not affected by sex [7]. However, no studies in Korea have reported differences in the relationship between SRH and low-grade inflammation according to sex. Therefore, in this study, using data from the 2015–2017 Korea National Health and Nutrition Examination Survey (KNHANES), we aimed to investigate the correlation between SRH and hs-CRP levels in Koreans aged ≥ 19 years and to analyze whether the correlation showed any differences between male and female subjects.

## Methods

### Participants

**Fig. 1:**
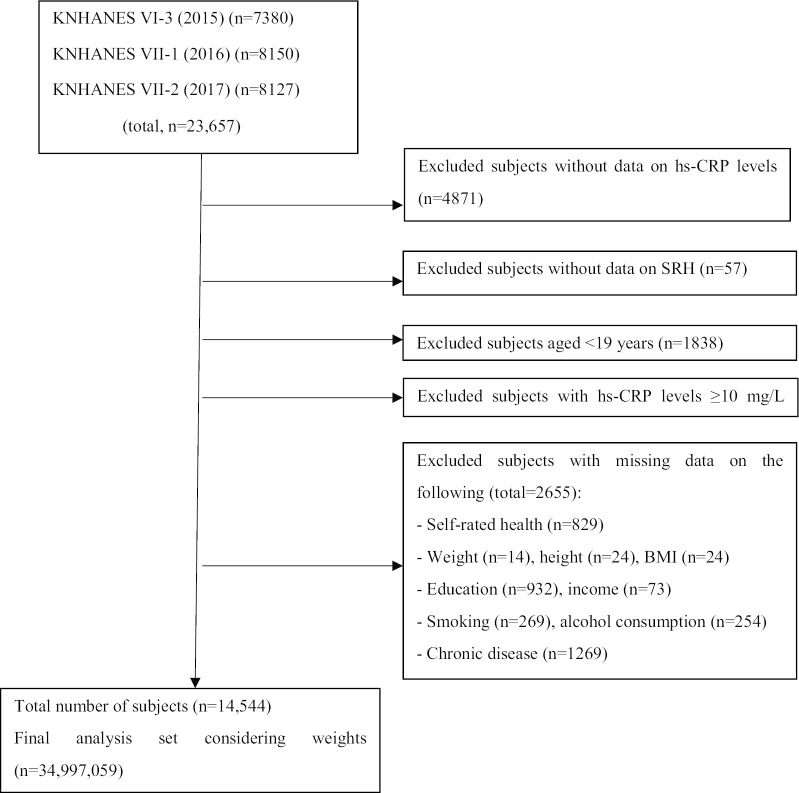
Flow diagram showing the number of included and excluded participants and the data for analysis. *KNHANES* Korea National Health and Nutrition Examination Survey, *hs-CRP* high-sensitivity C-reactive protein, *SRH* self-rated health, *BMI* body mass index

In this study, we used raw data from KNHANES VI and VII (2015–2017). KNHANES is a nationwide cross-sectional survey conducted by the CDC to ascertain the health and nutritional status of the Korean population. The survey extracts a representative sample of households and conducts a household member verification survey, health questionnaire survey, health examination, and nutritional survey for household members aged ≥ 1 year. In the present study, we aimed to investigate Korean adults aged ≥ 19 years. Of the 23,657 survey participants, we excluded individuals with missing measurements of hs-CRP (4871 people) and SRH (57 people), those aged < 19 years (1838 people), and those with unclear measurement values (2655 people). We also excluded individuals with hs-CRP levels ≥ 10 mg/L (866 people) as these levels could be considered to indicate acute infection, systemic inflammation, or tissue injury [15]. A total of 14,544 subjects (6281 men, 8263 women) were included in our analysis (Fig. 1).

### Instruments and procedure

#### SRH assessment

SRH was assessed using the question “In general, how would you rate your health?” There were five possible responses: “very good,” “good,” “fair,” “poor,” and “very poor.”

#### CRP measurement

hs-CRP was measured by immunoturbidimetry using a Cobas analyzer (Roche, Germany) and a Cardiac C-Reactive Protein High Sensitivity reagent (Roche, Germany). The minimum value in the specimens was 0.1 mg/L, and the maximum value was 20 mg/L. A high hs-CRP level was defined as an hs-CRP level > 1.0 mg/L.

The AHA and CDC recommend that an hs-CRP cutoff value of < 1 mg/L indicates low risk, 1–3 mg/L indicates average risk, and > 3 mg/L indicates high risk. However, these criteria were recommended based on studies focused on Western populations [16, 17]. As an alternative, we used an hs-CRP measurement greater than 1.0 mg/L as the cutoff value based on the studies by Jung et al. [18, 19].

#### Covariates

For the participants’ demographic characteristics, we included age; for socioeconomic characteristics, we included educational level, household income level, and marital status; for lifestyle factors, we included smoking status and alcohol consumption; and for disease and health-related factors, we included body mass index (BMI), chronic diseases, and menopausal status (women). Regarding chronic diseases, we included those directly or indirectly associated with hs-CRP: hypertension, diabetes mellitus, coronary artery disease (including myocardial infarction and angina [20]), dyslipidemia [21], stroke [22], and rheumatoid arthritis [23].

### Statistical methods

The KNHANES applies stratified cluster sampling and weighted values to a nationally representative sample, which is based on the reciprocal of selection probabilities (primary sampling unit, household), the inverse of the response rate (household, subject), and a post-stratification factor that provides age and sex-specific survey result estimates for the Korean population [24]. Therefore, we performed data analyses based on a complex sampling design with elements of stratification variables, clustering variables, and weights [25].

Data are presented as means ± standard errors for continuous variables or as frequencies and percentages for categorical variables. The following categorical variables were compared using the Pearson’s chi-square test: educational level, marital status, household income level, smoking status, alcohol consumption status, chronic diseases, SRH, and menopause. The following continuous variables were compared using the Mann–Whitney U test: age, weight, height, and BMI. Associations between hs-CRP levels and various sociodemographic categories and SRH were explored. In addition, multiple logistic regression analysis was used to study the relationship between hs-CRP levels and SRH according to sex. Results are presented as odds ratios (ORs) and 95% confidence intervals (CIs). Additionally, this study investigated if there was an underlying trend in the different levels of SRH in each model (Models 1–4) using Scheffe’s test after assigning an ordinal score to each SRH group in the model. A *P* value < 0.05 was considered significant. All data analyses were performed using the statistical software package SAS version 9.4 (SAS Institute Inc., Cary, NC, USA) and IBM SPSS Statistics 25 (SPSS Inc., Chicago, IL, USA).

## Results

### Characteristics of participants

**Fig. 2:**
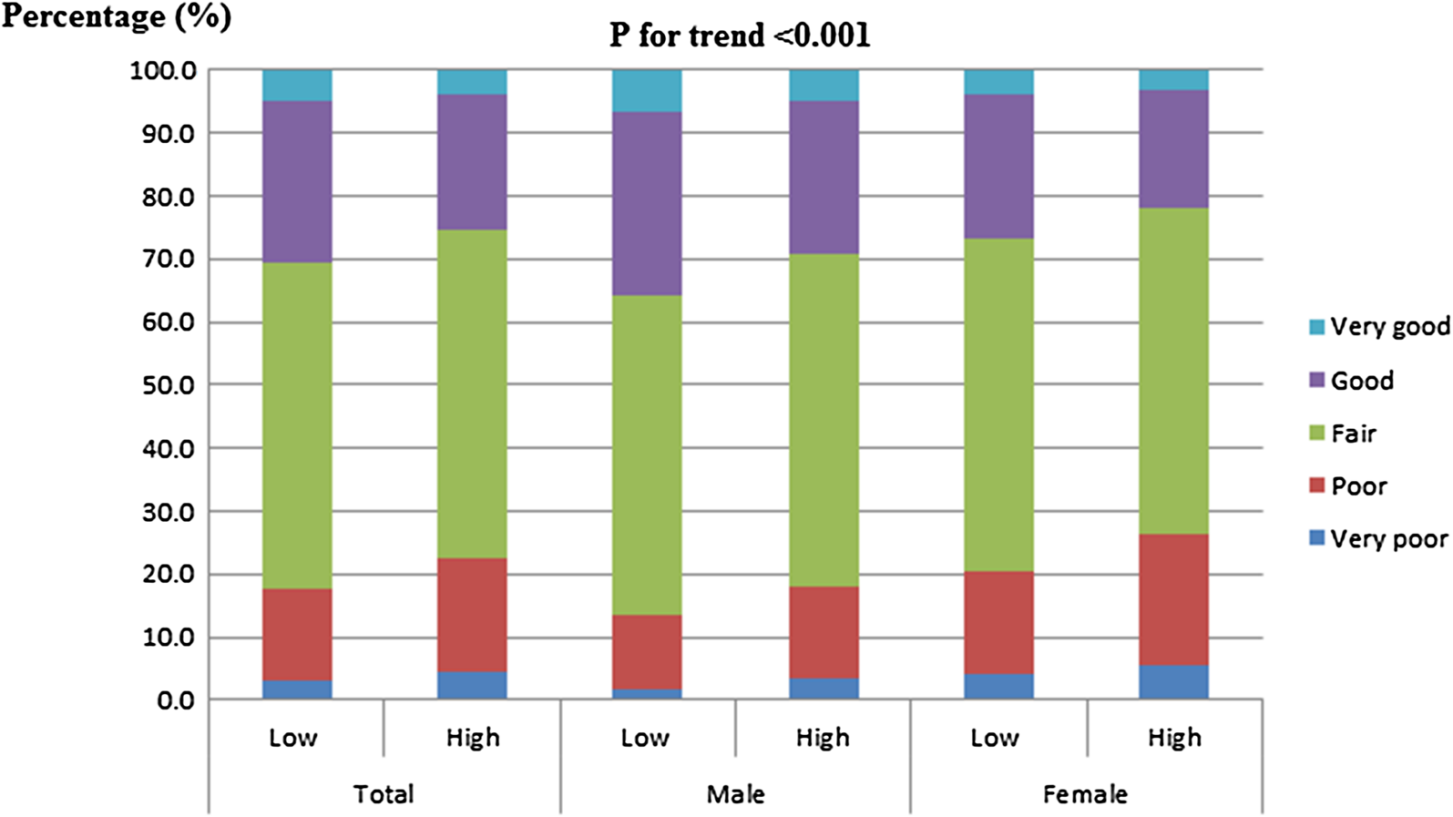
Prevalence of self-rated health (SRH) according to sex. The percentage of participants with very poor to poor SRH was higher in the group with high hs-CRP levels (22.47%) than that in the group with low hs-CRP levels (17.66%) (*P* < 0.001)

Table 1 shows the characteristics of the study participants (6281 men and 8263 women). To analyze the differences between the sexes, men and women were separately classified into groups according to their hs-CRP levels: low hs-CRP (≤ 1.0 mg/L) and high hs-CRP (> 1.0 mg/L). Among all participants, the percentage of those with very poor to poor SRH was higher in the high hs-CRP group (22.47%) than in the low hs-CRP group (17.66%). Similarly, for both men and women, the percentages of participants with very poor to poor SRH were higher in the high hs-CRP groups than in the low hs-CRP groups, whereas the percentages of participants with good to very good SRH were lower in the low hs-CRP groups than in the high hs-CRP groups (*P* < 0.001) (Fig. 2).

**Table 1:** Baseline characteristics of the study population in the 2015–2017 KNHANES

Characteristic	Men			Women		
	Low hs-CRP (≤ 1.0 mg/L) (n = 4495)	High hs-CRP (> 1.0 mg/L) (n = 1786)	*P* value^†^	Low hs-CRP (≤ 1.0 mg/L) (n = 6306)	High hs-CRP (> 1.0 mg/L) (n = 1957)	*P* value^†^
Age (years), mean (SD)	49.8 (0.249)	53.3(0.391)	.000*	49.9 (0.203)	54.2 (0.373)	.000*
Educational level, n (%)			.000*			.000*
Elementary school or lower	580 (12.9)	333 (18.6)		1424 (22.6)	653 (33.4)	
Middle school	446 (9.9)	205 (11.5)		651 (10.3)	203 (10.4)	
High school	1575 (35.0)	568 (31.8)		2001 (31.7)	547 (28.0)	
College or higher	1894 (42.1)	680 (38.1)		2230 (35.4)	554 (28.3)	
Marital status, n (%)			.002*			.000*
Married	3536 (78.7)	1487 (83.3)		5396 (85.6)	1772 (90.5)	
Unmarried	959 (21.3)	299 (16.7)		910 (14.4)	185 (9.5)	
Household income level, n (%)			.101			.009*
Low	1034 (23.0)	457 (25.6)		1444 (22.9)	522 (26.7)	
Lower middle	1077 (24.0)	464 (26.0)		1591 (25.2)	519 (26.5)	
Upper middle	1156 (25.7)	435 (24.4)		1609 (25.5)	470 (24.0)	
High	1228 (27.3)	430 (24.1)		1662 (26.4)	446 (22.8)	
Weight (kg), mean (SD)	70.0 (0.158)	73.4 (0.351)	.000*	56.8 (0.103)	62.1 (0.253)	.000*
Height (cm), mean (SD)	170.5 (0.101)	169.6 (0.161)	.000*	157.5 (0.081)	156.2 (0.149)	.000*
BMI (kg/m^2^), mean (SD)	24.0 (0.044)	25.4 (0.087)	.000*	22.9 (0.040)	25.4 (0.091)	.000*
Smoking, n (%)			.123			.483
Nonsmoker	1130 (25.1)	389 (21.8)		5710 (90.5)	1742 (89.0)	
Past smoker	1926 (42.8)	777 (43.5)		341 (5.4)	118 (6.0)	
Current smoker	1439 (32.0)	620 (34.7)		255 (4.0)	97 (5.0)	
Alcohol consumption, n (%)			.001*			.000*
Never drink	196 (4.4)	85 (4.8)		951 (15.1)	392 (20.0)	
< Once/month	1004 (22.3)	457 (25.6)		2634 (41.8)	864 (44.1)	
< Five times/month	1691 (37.6)	577 (32.3)		2020 (32.0)	496 (25.3)	
≥ Five times/month	1604 (35.7)	667 (37.3)		701 (11.1)	205 (10.5)	
Chronic diseases**, n (%)			.000*			.000*
None	2579 (57.4)	789 (44.2)		4000 (63.4)	966 (49.4)	
One	1186 (26.4)	590 (33.0)		1274 (20.2)	536 (27.4)	
Two or more	730 (16.2)	407 (22.8)		1032 (16.4)	455 (23.2)	
Self-rated health, n (%)			.000*			.000*
Very poor	80 (1.8)	59 (3.3)		270 (4.3)	108 (5.5)	
Poor	532 (11.8)	264 (14.8)		1026 (16.3)	410 (21.0)	
Fair	2271 (50.5)	943 (52.8)		3328 (52.8)	1009 (51.6)	
Good	1317 (29.3)	431 (24.1)		1431 (22.7)	370 (18.9)	
Very good	295 (6.6)	89 (5.0)		251 (4.0)	60 (3.1)	
Menopause, n (%)						.000*
No				3211 (50.9)	752 (38.4)	
Yes				3072 (48.7)	1189 (60.8)	

*KNHANES* Korea National Health and Nutrition Examination Survey, *SE* standard error, *SD* standard deviation, *BMI* body mass index

^*^p ≤ .05

^**^Chronic diseases include hypertension, diabetes, dyslipidemia, coronary heart disease (myocardial infarction or angina pectoris), stroke, and rheumatoid arthritis

^†^*P* values of *t* test or the Mann–Whitney U test for continuous variables and Pearson’s chi-squared test for categorical variables to determine differences between groups according to high-sensitivity C-reactive protein (hs-CRP) levels

### Correlation between SRH and high hs-CRP levels (overall)

Table 2 shows the overall correlations between SRH and high hs-CRP levels along with their respective ORs and 95% CIs. In the crude model (Model 1), the poorer the SRH, the higher was the risk in relation to the reference group (very good SRH). After adjustment for age, educational level, marital status, and household income levels (Model 2), the associations between fair to very poor SRH and high hs-CRP levels remained significant. However, after adjustment for BMI, smoking, and alcohol consumption (Model 3) and after additional adjustment for chronic diseases (Model 4), the associations only remained significant for poor and fair SRH.

**Table 2:** Association between high hs-CRP levels and SRH in all subjects

SRH	Model 1				Model 2				Model 3				Model 4			
	OR	95% CI		*P* value	OR	95% CI		*P* value	OR	95% CI		*P* value	OR	95% CI		*P* value
Very poor	2.02	1.47	2.78	.000*	1.54	1.11	2.12	.010*	1.22	0.87	1.72	.245	1.22	0.87	1.71	.259
Poor	1.82	1.44	2.29	.000*	1.68	1.32	2.13	.000*	1.40	1.09	1.79	.008*	1.38	1.08	1.77	.011*
Fair	1.51	1.22	1.87	.000*	1.46	1.18	1.82	.001*	1.34	1.06	1.68	.013	1.32	1.05	1.66	.016*
Good	1.16	0.92	1.46	.211	1.17	0.93	1.47	.183	1.12	0.88	1.42	.375	1.12	0.87	1.42	.377
Very good (ref.)	1.00				1.00				1.00				1.00			
P for trend	< 0.001*				< 0.001*				< 0.001*				< 0.001*			

Odds ratios with adjustments using logistic regression models. Model 1: unadjusted; Model 2: adjusted for age, educational level, marital status, and household income level; Model 3: adjusted for Model 2 confounders + body mass index, smoking, and alcohol consumption; Model 4: adjusted for Model 3 confounders + chronic diseases

*hs-CRP* high-sensitivity C-reactive protein, *SRH* self-rated health, *OR* odds ratio, *CI* confidence interval

^*^p ≤ .05

### Correlation between SRH and high hs-CRP levels (male vs. female participants)

When we analyzed male participants, those with fair to very poor SRH had a significantly higher risk of being in the high hs-CRP group than those with very good SRH in both the crude model (Model 1) and Model 2 (adjustment for age, educational level, marital status, and household income level). After adjustment for BMI, smoking status, and alcohol consumption status (Model 3), the associations between fair to very poor SRH and high hs-CRP levels remained significant. However, after adjustment for chronic diseases (Model 4), the association only remained significant for very poor and fair SRH (Table 3).

**Table 3:** Association between high hs-CRP levels and SRH in men

SRH	Model 1				Model 2				Model 3				Model 4			
	OR	95% CI		*P* value	OR	95% CI		*P* value	OR	95% CI		*P* value	OR	95% CI		*P* value
Very poor	2.69	1.68	4.32	.000*	2.19	1.37	3.51	.001*	1.78	1.07	2.96	.026*	1.74	1.04	2.90	.034*
Poor	1.79	1.30	2.44	.000*	1.70	1.23	2.35	.001*	1.43	1.02	2.00	.041*	1.40	0.99	1.97	.057*
Fair	1.63	1.22	2.16	.001*	1.61	1.20	2.14	.001*	1.44	1.06	1.96	.019*	1.42	1.04	1.93	.025*
Good	1.12	0.83	1.51	.462	1.14	0.84	1.54	.396	1.08	0.78	1.49	.637	1.08	0.78	1.49	.637
Very good (ref.)	1.00				1.00				1.00				1.00			
P for trend	< 0.001*				< 0.001*				< 0.001*				< 0.001*			

Odds ratios with adjustments using logistic regression models; Model 1: unadjusted; Model 2: adjusted for age, educational level, marital status, and household income level; Model 3: adjusted for Model 2 confounders + body mass index, smoking, and alcohol consumption; Model 4: adjusted for Model 3 confounders + chronic diseases

*hs-CRP* high-sensitivity C-reactive protein, *SRH* self-rated health, *OR* odds ratio, *CI* confidence interval

^*^p ≤ .05

When we analyzed female participants, those with fair to very poor SRH had a significantly higher risk of being in the high hs-CRP group than those with very good SRH in the crude model (Model 1). After adjustment for age, educational level, marital status, and household income levels (Model 2), the association only remained significant for poor SRH. However, in contrast with that observed for male participants, no significant correlations remained after adjustment for BMI, smoking, and alcohol consumption (Model 3) and after additional adjustment for chronic diseases (Model 4) (Table 4).

**Table 4:** Association between high hs-CRP levels and SRH in women

SRH	Model 1				Model 2				Model 3				Model 4			
	OR	95% CI		*P* value	OR	95% CI		*P* value	OR	95% CI		*P* value	OR	95% CI		*P* value
Very poor	1.97	1.28	3.01	.002*	1.35	0.87	2.10	.182	0.99	0.62	1.59	.980	1.01	0.63	1.62	.968
Poor	2.03	1.43	2.90	.000*	1.77	1.24	2.53	.002*	1.39	0.95	2.03	.086	1.40	0.96	2.03	.083
Fair	1.50	1.06	2.11	.021*	1.38	0.98	1.95	.063	1.27	0.89	1.80	.186	1.27	0.89	1.80	.187
Good	1.25	0.88	1.78	.213	1.24	0.87	1.76	.233	1.21	0.84	1.76	.301	1.22	0.84	1.76	.297
Very good (ref.)	1.00								1.00				1.00			
P for trend	< 0.001*				< 0.001*				< 0.001*				< 0.001*			

Odds ratios with adjustments using logistic regression models; Model 1: unadjusted; Model 2: adjusted for age, educational level, marital status, and household income level; Model 3: adjusted for Model 2 confounders + body mass index, smoking, alcohol consumption, and menopause; Model 4: adjusted for Model 3 confounders + chronic diseases

*hs-CRP* high-sensitivity C-reactive protein, *SRH* self-rated health, *OR* odds ratio, *CI* confidence interval

^*^p ≤ .05

## Discussion

Our findings were similar to those of previous studies that reported a correlation between poor SRH and a high hs-CRP level [7–9, 26]. In a study of 4049 respondent older adults without significant cognitive deficit by Szybalska et al. [27], a worse SRH was associated with increased interleukin-6 (IL-6) and CRP levels. Leshem-Rubinow et al. [7] analyzed the correlations between SRH and the inflammation-sensitive biomarkers hs-CRP and fibrinogen in 13,773 healthy individuals and observed higher biomarker levels in the group with the lowest SRH level; hs-CRP showed a correlation in both men and women, but fibrinogen only showed a correlation in men. Shanahan et al. [8] studied 13,236 young adults and reported that on adjusting for acute/chronic diseases, medication history, and health behaviors, a lower SRH level was associated with a higher hs-CRP level; however, on adjusting for BMI, the correlation in female participants was weakened, whereas the correlation in male participants remained significant. However, a study of 16,256 Japanese individuals reported a significant correlation between poor SRH and a high hs-CRP level only in female participants [9]. Thus, while correlations between poor SRH and a high hs-CRP level have been reported, the above studies showed limitations such as restricted age of participants, the inclusion of only older adults [26] or only young adults [8], and the lack of consideration for diseases that could affect the relationship between SRH and CRP [9]. Moreover, there have been few studies on the relationship between SRH and CRP [7, 28] so far; there have been no such studies in Koreans.

Several studies have reported correlations between poor SRH and pro-inflammatory cytokines, including IL-6 [17, 29]. CRP is produced by hepatocytes under the regulatory control of IL-6 and other inflammatory cytokines [30], and these pro-inflammatory cytokines cause sickness behaviors, such as weakness, depression, exaggerated pain (hyperalgesia or allodynia), and lack of appetite [31]. In other words, the relationship between poor SRH and a high hs-CRP level can be explained by differences caused by pro-inflammatory cytokines, and this, in turn, can explain our results.

Inflammatory indices in women are known to be altered by the menstrual cycle, menopause, and hormone therapy [32], and menopause and estrogen replacement therapy have been reported to affect obesity and inflammation in women [33]. CRP levels can be presumed to change depending on the hormonal environment, and this could act as a confounding factor in the relationship between SRH and hs-CRP [34]. In our study, we were unable to investigate whether participants were taking female hormones or their stage in the menstrual cycle. As we only accounted for female menopause, we may not have observed significant results for the association between SRH and hs-CRP levels in women. Moreover, biological sex is known to affect CRP-related genetic variation [35], and according to a study by Kettunen et al. [36], allelic variants in the CRP gene are associated with CRP levels, and men and women show differences depending on the CRP genotype. Hence, the differences in genetic variation between men and women could have affected our results.

Sex differences have been reported in the relationship between CRP levels and mortality [37–40], but it is unclear why a high CRP level is only associated with an increased mortality risk in men. Dong et al. analyzed the middle-aged Chinese population; hs-CRP was associated with increased risk of developing CVD [41]. In addition, Lee JH et al. [42] studied 23,233 rural Koreans and reported that a high CRP level was more strongly associated with higher mortality in men than in women. There have been several studies reporting a stronger correlation between SRH and mortality in men than in women [40, 43]. Specifically, men with poor SRH have been reported to show a higher risk for conditions related to mortality, such as CVD and cancer. When assessing SRH, the subject rates their current overall health; it has been reported that men rate their own health in comparison to that of other men, and male SRH tends to mostly reflect serious and life-threatening disease, whereas female SRH tends to reflect other factors unrelated to mortality and chronic, non-life-threatening disease, resulting in a weaker correlation between SRH and mortality for women [43–45]. Moreover, in a study of Korean adults by Shin et al. [46], women tended to rate their own health more poorly than men, and in a study by Lee SY et al. [47], traditional Korean gender roles had a negative effect on women, and the risk of poor SRH was higher among Korean women than among women from the US. Similarly, in our study, we only observed a correlation between SRH and hs-CRP among male participants. The discrepancy between men and women could be related to the fact that CRP is more likely to reflect CVD and mortality in men than in women and that SRH is more likely to directly reflect health and mortality in men than in women.

In our study of Korean adults aged ≥ 19 years, when we analyzed all participants, those in the poor SRH group were more likely to have high hs-CRP levels (> 1.0 mg/L) than those in the very good SRH group. Especially in male participants, as SRH changed from very good to very poor, there was a corresponding increase in the risk of high hs-CRP levels (> 1.0 mg/L). These results can be explained by the fact that immune-related activity is associated with vague symptoms of malaise and interoceptive perception [29]. Such findings are consistent with those of a previous study of healthy adults, in which poorer SRH was associated with increased serum inflammatory marker levels (IL-6 and CRP) [26].

Even after correcting for all sociodemographic characteristics, health-related factors, and chronic diseases known to be associated with low-level inflammation, among male participants, the very poor SRH group showed a 1.74-times higher risk of high hs-CRP levels than the very good SRH group, but there was no significant relationship among female participants. This finding could be because SRH is a dynamic evaluation for judging the trajectory of health, which reflects both clinical stage and preclinical stage disease [48]. Therefore, even after correcting for chronic diseases associated with hs-CRP, we still observed a correlation in male participants.

Our study has several limitations. First, because this was a cross-sectional study, it was not possible to infer causal relationships, and we could only investigate the correlation between SRH and hs-CRP. Nevertheless, the value of this study is that we used data from the KNHANES, which is representative of the Korean population, and that it was a large-scale study of Korean adults. Second, SRH assessment was performed at specific times. Future monitoring studies are necessary to ascertain the long-term relationships between SRH and hs-CRP. Third, we only used hs-CRP as an inflammatory marker; further studies will need to investigate the correlations of SRH with other indicators (e.g., IL-6, tumor necrosis factor-alpha). Finally, because the study was based on data from a survey of South Koreans, the results could have been affected by the racial characteristics of Koreans and may thus be difficult to apply to people of other races. Despite these limitations, our study showed a strong correlation between poor SRH and a high hs-CRP level in Korean male adults and is valuable as the first study to examine the relationship between SRH and hs-CRP in Korean adults.

## Conclusions

We investigated the relationship between SRH and hs-CRP levels through a survey of Korean adults. Even after correcting for factors that could affect low-grade inflammation, such as age, socioeconomic status, BMI, health-related behaviors, and chronic diseases, male adults with poor SRH were at a greater risk of having high hs-CRP levels. This study showed a close relationship between low-grade inflammation (high hs-CRP levels) and SRH, an instrument reflecting one’s own assessment of their health. We only observed a correlation in male participants; this suggests that there could be factors that affect the relationship between SRH and hs-CRP differently in men and women. Our findings could provide a basis for developing health improvement programs. Future studies will need to be conducted to examine sex differences in the relationship of SRH with other inflammatory markers.

## Data Availability

All original data are publicly available free of charge from the KNHANES website (https://knhanes.cdc.go.kr) for the purposes of academic research.

## Abbreviations

AHA: American Heart Association
BMI: Body mass index
CDC: Centers for Disease Control
CI: Confidence interval
CVD: Cardiovascular disease
hs-CRP: High-sensitivity C-reactive protein
KNHANES: Korea National Health and Nutrition Examination Survey
OR: Odds ratio
SRH: Self-rated health
